# Deficiency in homozygous haplotypes reveals recessive lethal variants affecting fertility and viability in the Friesian horse

**DOI:** 10.1186/s12864-026-12728-5

**Published:** 2026-03-11

**Authors:** Marije J. Steensma, Bart J. Ducro, Harmen P. Doekes, Bert Dibbits, Martien A. M. Groenen, Martijn F. L. Derks

**Affiliations:** 1 Animal Breeding and Genomics, Wageningen University and Research, Wageningen, the Netherlands; 2 Koninklijke Vereniging “het Friesch Paarden-Stamboek”, Drachten, the Netherlands

**Keywords:** Lethality, *Equus caballus*, Homozygous deficient haplotypes, Causal variant, Friesian horse, Fertility, Risk matings, Genotype data, Whole genome sequencing

## Abstract

**Background:**

Recessive lethal alleles causing pre- or postnatal death in homozygous mutant animals, could lead to reduced fertility success. The Friesian horse breed has signs of reduced fertility and has faced high inbreeding rates in the past (∆F > 1%). Consequently, by genetic drift lethal alleles may have reached moderate to high frequencies in the population. Our aim was to identify lethal recessive alleles that — when homozygous — may cause pre- or postnatal death in the Friesian horse.

**Results:**

We analyzed genotypes (70 K SNP) of over 8,000 Friesian horses, looking for haplotypes with a homozygous deficiency, and used available sequence data of 50 Friesian sires to pinpoint the likely causal variant. A deficit in homozygous animals suggests a lethal allele, because individuals inheriting two copies of such an allele likely die before birth or die before being genotyped, creating a detectable imbalance in genotype frequencies. We found ten candidate haplotypes in the Friesian horse with carrier frequencies ranging from 8.0 to 22.1%. We identified candidate causal variants of six haplotypes, of which two were associated with the already known genetic disorders dwarfism and hydrocephalus. The other candidate variants were a 261-kilobase-pair deletion affecting several non-coding RNA’s, and a 14-base-pair frameshift deletion in the *MET* gene. Three haplotypes in LD comprised the deletion in *MET* and were associated with a 25% reduction (*P* < 0.001) in insemination success in risk matings, likely caused by early embryonic lethality.

**Conclusions:**

In general, considering the population characteristics of domestic horse breeds, we strongly recommend performing such analyses in other horse breeds using the increasingly available genotype data. Such analyses could provide important contribution to the improvement of fertility rates in horse populations.

**Supplementary Information:**

The online version contains supplementary material available at 10.1186/s12864-026-12728-5.

## Background

Horse breeds are characterized by selective breeding programs, which has led to high inbreeding rates and a small effective population size (Ne) in several horse populations [1–6]. While selection removes deleterious alleles over time, in populations with small Ne, the effects of genetic drift may outweigh the effects of selection. Thus, in such populations, recessive lethal mutations can increase in frequency. At a certain frequency, there is a trade-off between genetic drift and selection, preventing further increase in frequency of those recessive lethal alleles due to the loss of homozygotes. Still, those recessive lethal alleles can reach up to 20% carrier frequency by genetic drift alone, thereby increasing the chance of carrier-by-carrier matings, resulting in a 25% chance in homozygous animals expressing the lethal phenotype [7].

Recessive lethal alleles can cause pre- or postnatal lethality in homozygous mutant animals. Phenotypic detection depends on the stage at which the recessive lethal allele causes lethality. For example, recessive alleles that cause lethality at a later stage of gestation or in early life result in clear phenotypes. This has led to the identification of several lethal alleles causing stillbirths [7–10], death shortly after birth [11], or during rearing [9, 12, 13]. However, recessive lethal alleles that cause death during the first three weeks after fertilization are often only reflected by a reduced insemination success and normally go undetected.

The use of large-scale genotype data has already led to the identification of several lethal mutations in livestock species, including those causing embryonic lethality [7, 9, 11, 14, 15]. The approach developed by VanRaden et al., (2011) [14] uses genotypes to identify haplotypes with a deficit in homozygous animals, indicating regions which may harbor recessive lethal alleles. Such studies on lethal mutations are of particular interest in domestic horse populations because of small Ne and relatively low per cycle pregnancy rates (~ 65%) in horses [16–19]. However, due to the lack of large-scale genotype data in horses, only one study to date has reported embryonic lethal alleles in horses [20].

Since 2023, genotypes have been routinely collected for Friesian horses as part of the registration process for foals, allowing for a scan for recessive lethal alleles in the Friesian horse population. The Friesian horse population has a closed population status, a popular sire effect and a small Ne (currently < 100, historically < 50) [6]. In the Friesian horse, the causal mutations for the genetic disorders dwarfism and hydrocephalus have already been identified both with carrier frequencies of ~ 14%. In addition, there are signs of reduced fertility in Friesian horses [4]. Due to its population characteristics, it is therefore likely that there are more yet unidentified lethal alleles segregating at moderate frequencies in the Friesian horse. Thus, the Friesian horse provides a highly relevant case for the identification of such recessive lethal alleles.

Here, we aim to identify recessive lethal alleles segregating that may cause pre- or postnatal lethality in the Friesian horse. We scanned the population for deficit homozygous haplotypes and used available sequence data of Friesian sires to pinpoint the likely causal variant. In addition, we studied the effect of the identified haplotypes on insemination success, still birth rate, and juvenile mortality.

## Methods

### Ethics statement

Samples collected for DNA extraction were only used for routine diagnostic purposes of the breeding program, and not specifically for the purpose of this project. Therefore, no ethical approval was required for this study. Sample collection was conducted strictly according to the Dutch law on animal protection and welfare (Wet Dieren).

### Animals, genotypes and filtering

The dataset consists of 8,247 genotyped Friesian horses born between 1996 and 2024. The animals are genotyped on the GGP Equine 70 K Plus BeadChip (Neogen/Illumina) with 71,781 SNPs. Pedigree information was retrieved from “Het Koninklijk Friesch-Paarden Stamboek” (KFPS). The SNP array used in the initial genotyping analysis was developed based on chromosomal positions of the EquCab3.0 reference assembly. The genotype data were lifted over to the EquCab_Friesian_WUR reference genome (GenBank accession: GCA_964023265.1) using Crossmap v.0.7.3 [21] with probe sequence information. In total, 6,814 SNPs did not map to EquCab_Friesian_WUR and 1,085 SNPs that mapped on multiple positions on EquCab_Friesian_WUR were excluded. Additionally, duplicate SNPs and SNPs mapped to contigs were removed, and only autosomal SNPs were kept, resulting in a dataset of 63,831 markers. Subsequently, SNPs and animals were filtered using Plink v.1.9 [22] on the following criteria: (i) minor allele frequency > 1%, (ii) genotype call frequency > 90%, (iii) animal call frequency > 70%, and no highly significant deviation from the Hardy-Weinberg equilibrium (*P* < 10^− 8^). After quality control, the final dataset consisted of 40,809 markers (Additional file 2: Table S1).

### Whole genome sequence data and mapping

Whole Genome Sequence (WGS) data was available of 50 Friesian horses from paired-end 150 bp reads sequenced by BGI Genomics on DNBSEQ-T7 (mean sequencing depth = 24.9X, range 12.8–50.0X). The sequenced horses were approved breeding sires, born between 1996 and 2018, that were pre-selected for sequencing by the KFPS using marginal contributions to capture as much as possible of the genetic variation present in the Friesian horse population. In total, the current number of approved breeding sires in the Friesian horse studbook is around 100. Thus, sequence data of ~ 50% of all approved breeding sires is available. Most of the sequenced animals also had data from the GGP Equine 70 K Plus BeadChip (*N* = 34). WGS data was aligned to both the EquCab_Friesan_WUR reference genome (GenBank accession: GCA_964023265.1) and EquCab3 (GCA_002863925.1) using BWA-MEM2 v2.2.1 [23]. SAMBLASTER v.0.1.26 [24] was used to mark duplicates and SAMtools v1.17 [25] to sort and index the BAM files.

### Pre-processing

For some sires (*N* = 16) we had WGS data but no data from the GGP Equine 70 K Plus BeadChip. For these sires, genotype information of the filtered 40,809 markers was extracted from the final variant call format (VCF) file generated with Freebayes variant caling software [26] (see methods: variant calling, filtering and annotation). Then, the program conform-gt version 24May16.cee [27] was run to make the markers from the WGS data consistent with the genotype data (reference panel). Subsequently, markers from the WGS data of the sires (*N* = 16) were merged with the available genotype data of the genotyped horses (*N* = 8,247). As a result, 40,809 SNPs from 8,263 horses were available for subsequent analysis (based on EquCab_Friesian_WUR). Finally, genotypes were phased using BEAGLE version 5.3 [43].

### Identification of homozygous haplotype deficiency

To identify haplotypes that carry recessive (semi-)lethal alleles, the phased haplotypes from 8,263 horses were analyzed for complete absence or a reduced level of homozygosity. Haplotypes were identified using an overlapping sliding window approach of 1 and 2 Mb. We used fixed 1 and 2 Mb windows to account for variable LD structure across the genome, and we interpreted adjacent significant windows as representing the same underlying region. Within each window, individual haplotypes (with a frequency of > 0.5% to avoid haplotypes that appear due to error in phasing and haplotypes that provide insufficient statistical power to investigate associated phenotypes or potential causal mutations) were evaluated for missing or deficit homozygosity with a maximum observed homozygosity of < = 30 animals. The expected number of homozygotes was estimated based on haplotype frequency using the Hardy-Weinberg principle. An exact binomial test was applied to test the number of observed homozygotes with the number of expected homozygotes. Haplotypes were considered significant if the Bonferroni-adjusted *P*-value was < 0.05.

### Phenotypic effects associated with homozygous deficient haplotypes

To study the association between the homozygous deficient haplotypes and the causal phenotype, breeding and foal registration data was studied. Data of all matings between 1999 and 2022 (*N* = 224,292) and data of all offspring born between 2000 and 2023 (*N* = 117,139) was retrieved from the KFPS. Based on those records, three phenotypes were evaluated: insemination success, stillbirth rate and juvenile mortality. Insemination success was defined as the % of all matings (excluding re-matings within 10 days) that resulted in a foal (dead or alive) reported to the KFPS. Stillbirth rate was defined as the % of foals were reported to the KFPS as dead-born or died on the day of birth. Juvenile mortality was defined as the % of foals that died within the first 365 days including all cases of death.

**Fig. 1:**
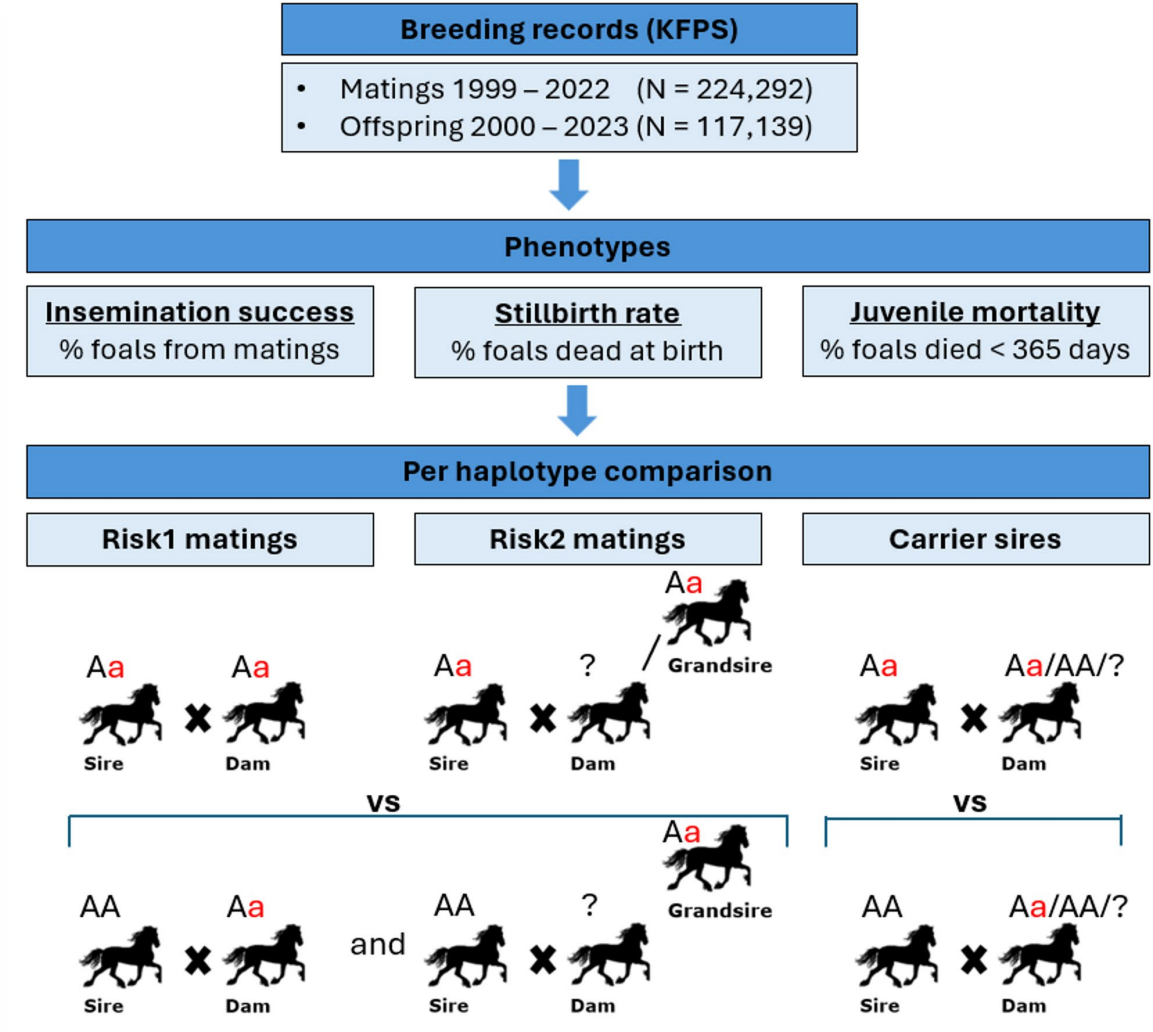
Overview of the phenotypic analysis for candidate lethal haplotypes. Mating records and offspring records were retrieved from the KFPS. From those records, the phenotypes insemination success, stillbirth rate and juvenile mortality could be retrieved. For each haplotype separately, differences in phenotypes were analyzed by comparing phenotypes of risk1 matings and risk2 matings to matings of non-carrier sires with carrier dams or carrier maternal grandsires. Additionally, differences in phenotypes were analyzed by comparing all matings from carrier sires to all matings from non-carrier sires

To investigate the impact of the candidate lethal haplotypes on these phenotypes, differences in phenotypes were analyzed separately for each haplotype using three mating categories (Fig. 1). The first mating category, were risk1 matings comprising matings between carrier sires x carrier dams. Second, as only a few current breeding dams have already been genotyped (*N* < 2,500), we also analyzed risk2 matings which consisted of matings between carrier sires x carrier maternal grandsires. If the maternal grandsire was carrier, but the dam was genotyped and non-carrier, then this mating was not included. Also, if the dam was genotyped and carrier, this dam was only included in risk1 and not in risk2 matings. Phenotypes of risk1 and risk2 matings were compared to non-risk matings comprising non-carrier sires (genotyped) with the same group of dams as defined in risk1 and risk2 matings. Lastly, to increase the number of matings in the phenotypic analysis, we also compared phenotypes of all matings of carrier sires (independent of the carrier or genotype status of the dam) with all matings of non-carrier sires. For each mating category, all available data were used to calculate the corresponding phenotype at the haplotype and mating category level. To test whether two groups significantly differed from each other, we applied a two-tailed proportion z-test.

### Effect on semen quality

The candidate variant for FH10 is missing some ncRNAs likely associated with spermatogenesis (see results section). Therefore, we tested whether carrier sires for FH10 had lower semen quality parameters compared to non-carrier sires. In total, semen reports of 81 approved sires which were sequenced or genotyped (70 K SNP) were available. Semen quality parameters were not normally distributed and therefore the median was calculated between carriers and non-carriers of a haplotype. Sires were defined as carrier if the candidate variant was found in their sequence data, or if they were haplotype carrier based on the genotype data. A Wilcoxon Rank Sum test was performed to test whether there was a significant difference between the median of carries and non-carriers (*P* < 0.05).

### Variant calling, filtering and annotation

To pinpoint the candidate variants causing the homozygous deficient haplotypes, both single nucleotide polymorphisms (SNPs), indels and structural variants were called using the available whole genome sequence data.

#### Single nucleotide polymorphisms and indels

Freebayes variant caling software [26] was used to call variants (SNPs and indels) in all 50 sequenced sires with WGS data mapped to both EquCab_Friesian_WUR and EquCab3, with the following settings: --use-best-n-alleles 4 –min-base-quality 10 –min-alternate fraction 0.2 –haplotype-length 0 –ploidy 2 –min-alternate count 2. In addition, variants with a low phred quality score (< 20) were discarded. Variant annotation was done with BCFtools/csq [28]. The impact of missense variants was predicted using sorting intolerant from tolerant (SIFT) [29] based on the position on EquCab3 if the variant was known in Ensembl, or on the position on EquCab_Friesan_WUR if the variant was not known. In addition, for variants in a conserved splice region between human and horse, combined annotation dependent depletion (CADD) (v1.7) [30] scores were derived from the UCSC human genome browser [31]. The following variant classes were considered potentially causing loss-of-function (LoF): splice acceptor, splice donor, inframe indels, frameshift, stop loss, stop gained, start lost variants.

#### Structural variation

The Smoove pipeline (https://git.wur.nl/kim.lensing/population-structural-var-calling-smoove-version2) was used to call and annotate structural variants (SVs) in all 50 sires with WGS data. Smoove (v0.2.8) uses various software to call and filter SVs taking the aligned BAM files and the reference genome as input. First, Lumpy (v0.3.1) was used to call SVs. Lumpy uses signals from split reads and discordant paired end reads to predict breakpoints of deletions, duplications and inversions [32]. Then, SVtools (v0.4.0) [33] was used to combine all SV calls into one single variant call format (VCF) file. Next, all samples were genotyped for population-wide non-redundant SV sites with SVTyper (v0.7.0) [34]. As a result, the Smoove pipeline generated a VCF file in which all detected SVs were denoted, and each sample was assigned a genotype for each SV. SVs which comprised at least one (or a part of a) coding region (exon) were considered to cause LoF. To minimalize the number of false negatives, no further filtering was performed.

### Identification of candidate causal variants

We looked for variants called against the reference genome EquCab_Friesian_WUR. However, the Friesian horse used to develop this reference genome was also sequenced and included in the analysis. Therefore, if for a certain haplotype the Friesian horse of the reference genome was carrier himself, also variants against EquCab3 were analyzed.

We identified candidate causal variants for the haplotypes exhibiting missing homozygosity, using the following criteria: (i) the variant was located within 4 Mb of the haplotype boundaries, (ii) all sequenced haplotype carrier sires were heterozygous for the variant and a maximum of one homozygous sequenced sire was observed (allowing for one false genotype assignment), and (iii) a maximum of three sequenced non-haplotype-carrier sires carried the variant.

From the remaining candidate causal variants, the LoF, missense and splice region variants were selected as most likely candidates. Of these most likely candidates, we retrieved information from several databases: (i) the SIFT score [29] for missense variants, (ii) the conservation score of the variant, and (iii) whether the variant is known, its frequency, and if it occurs in homozygous state based on the European Nucleotide Archive. In total, whole genome sequence data of 94 horses are available in The European Nucleotide Archive under project accession numbers PRJEB28306 and PRJEB9799. This publicly available data does not contain sequence data of Friesian horses. To predict the relative impact on the phenotype, further functional support in the form of phenotypes from null-mutant mice and gene expression was obtained from the MGI database release 6.24 [35].

### PCR validation of two candidate variants

To further validate the candidate variantvariants for FH2/3/4 and FH10, we tested the carrier state of those variants in the animals that were found to be homozygous for those haplotypes based on the 70 K SNP data. We were able to collect hair samples of four animals (F1-4) homozygous for FH10 and of two animals (F5-6) homozygous for FH3. Genomic DNA was isolated from hair roots of these six animals with the nucleospin tissue kit from Bioke (support protocol “hair roots”). Quality of gDNA was checked on Denovix and Qubit (Additional file 2: Table S2) and on 0.8% agarose gel, with 40 V and three hours run time (Additional file 1: Fig. S13). Primers were designed with primer 3 (https://primer3.ut.ee/) (Additional file 2: Table S3). The PCR cycling conditions were 95 °C for 5 min; 35 cycles of 30 s at 95 °C, 45 s at 55 °C annealing temperature, 90 s at 72 °C; followed by an elongation step of 72 °C for 10 min and thereafter kept at 10 °C. The PCR products for the 14 bp deletion chromosome 4 were diluted 250X in H2O (MQ water). Fragment analysis was performed on an ABI3730, with diluted PCR products in formamide with marker LIZ500. The PCR products for the 261 kb deletion were put on 2% agarose gel (2 µl PCR product, 6 µl H2O MQ water, 2 µl BFB loading buffer). PCR products were run on this gel for 1,5 h at 120 V.

### RNA-seq data

The gene expression in a 261-Kb deleted region was assessed using RNA-seq data. There was RNA-seq data from three immature and three mature testes of six Mongolian horses available in the online Sequence Read Archive (SRA) under Bioproject PRJNA395221. The RNA-seq data of the six testes were mapped to the EquCab_Friesian_WUR reference genome using Hisat2 (v2.2.1) [36] and further visualized in JBrowse [37]. Stringtie (v2.2.3) [38] was run to get FPKM (fragments per kilobase per million fragments mapped) and TPM (transcripts per kilobase million) values. FPKM values normalize for gene length and sequencing depth, enabling gene count comparisons between genes of the same sample. TPM values count the transcripts (kb) per million reads mapped, enabling gene count comparisons between samples of the same sample group.

## Results

### Detection of lethal and semi-lethal recessive haplotypes segregating at moderate frequencies

Analysis for missing homozygotes on genotype data of 8,263 Friesian horses yielded 10 significant (Bonferroni adjusted) haplotypes (denoted FH1 – FH10) with carrier frequences ranging from 8.0% to 22.1% (Table 1; Additional file 2: Table S1 and S4). No homozygotes were observed for the haplotypes FH1, FH4, FH5, and FH8 while at least 38, 15, 16, and 34 were expected, respectively. Homozygotes were observed for the haplotypes FH2, FH3, FH6, FH7, FH9, and FH10, possibly suggesting semi-lethal variants or incomplete linkage disequilibrium of the haplotypes with the causal lethal recessive variant (Table 1). The haplotypes FH2, FH3, and FH4 were all located on chromosome 4 and were in linkage disequilibrium (LD), sharing partly the same carriers (Additional file 1: Fig. S1), suggesting they comprise the same causal variant. The haplotypes FH1 and FH8 comprised regions of the known genetic disorders hydrocephalus [8] and dwarfism [12], respectively.

**Table 1:** Haplotypes with homozygous deficiency. The 10 haplotypes with homozygous deficiency based on EquCab_Friesian_WUR reference genome with Bonferroni adjusted *P* < 0.05. In total, 70 K SNP Chip data of 8,263 horses were included in the analysis. The table shows the genomic location, carrier frequency, and the deficit of homozygosity for each haplotype. This deficit of homozygosity is based on haplotype frequency, using the Hardy-Weinberg principle

Hap.	Chr	Start (Mb)	End (Mb)	Carrier freq.	Homozygous animals		
					Obs.	Exp.	*P*_adj
FH1	1	74	81	12.8%	0	38	2.37E-11
FH2	4	67.5	69.5	14.7%	15	53	5.36E-05
FH3	4	71	73	13.7%	5	45	1.28E-09
FH4	4	79	81	8.0%	0	15	3.06E-02
FH5	5	49	51.5	8.4%	0	16	9.48E-03
FH6	6	53	56	22.1%	23	132	9.97E-27
FH7	9	63	65.5	16.7%	20	61	6.93E-03
FH8	14	8	11	11.7%	0	34	2.12E-09
FH9	18	18	20	11.7%	1	32	2.16E-08
FH10	23	9	12	16.5%	5	68	2.23E-18

### Risk matings and matings from carrier sires show lower insemination success, more still births and higher juvenile mortality

**Fig. 2:**
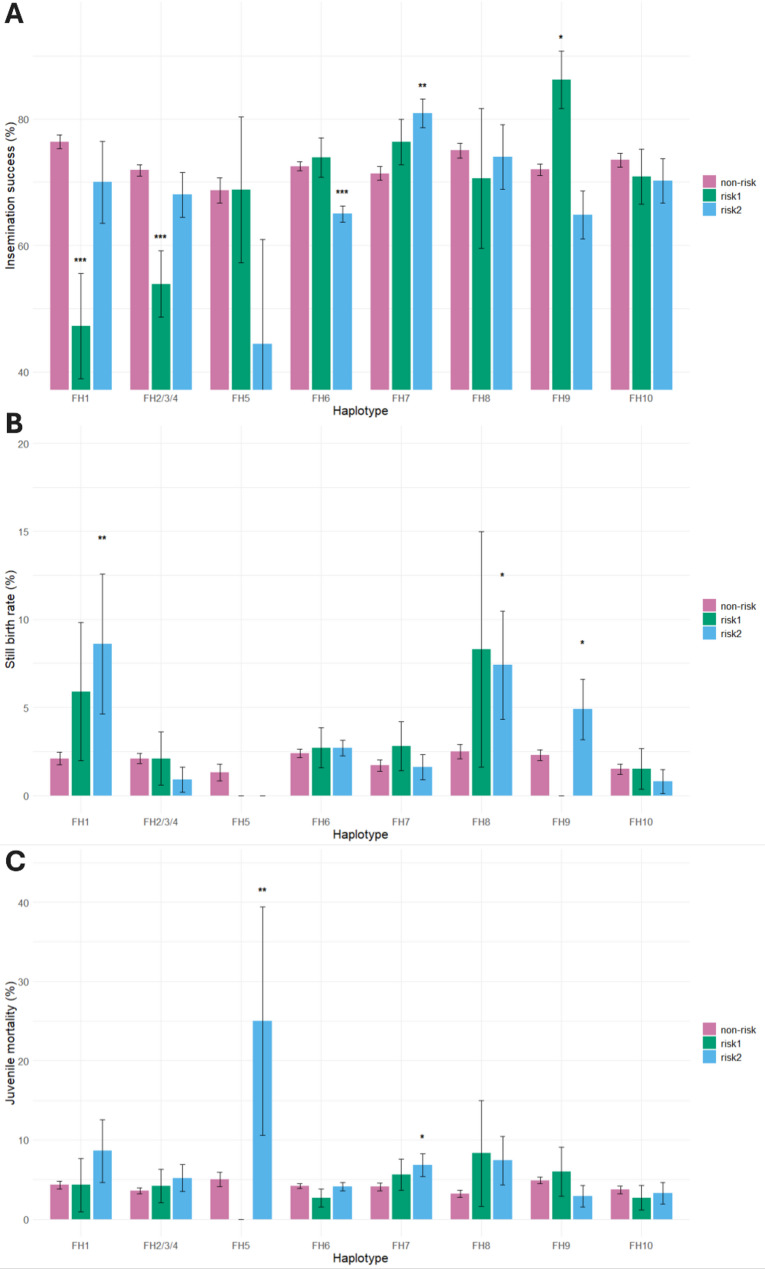
Insemination success, still birth rate and juvenile mortality of non-risk, risk1, and risk2 matings, for each haplotype. **A** Insemination success of risk versus non-risk matings. **B** Still birth rate of risk matings compared to non-risk matings. **C** Juvenile mortality of risk matings compared to non-risk matings. Risk1 matings were defined when both the sire and dam were carriers. Risk2 matings were defined when the sire was carrier and the maternal grandsire was carrier (dam status was unknown). Non-risk matings were defined when the sire was non-carrier and the dam was carrier or maternal grandsire was carrier (dam status was unknown). *P < 0.05, **P < 0.01, ***P < 0.001

**Fig. 3:**
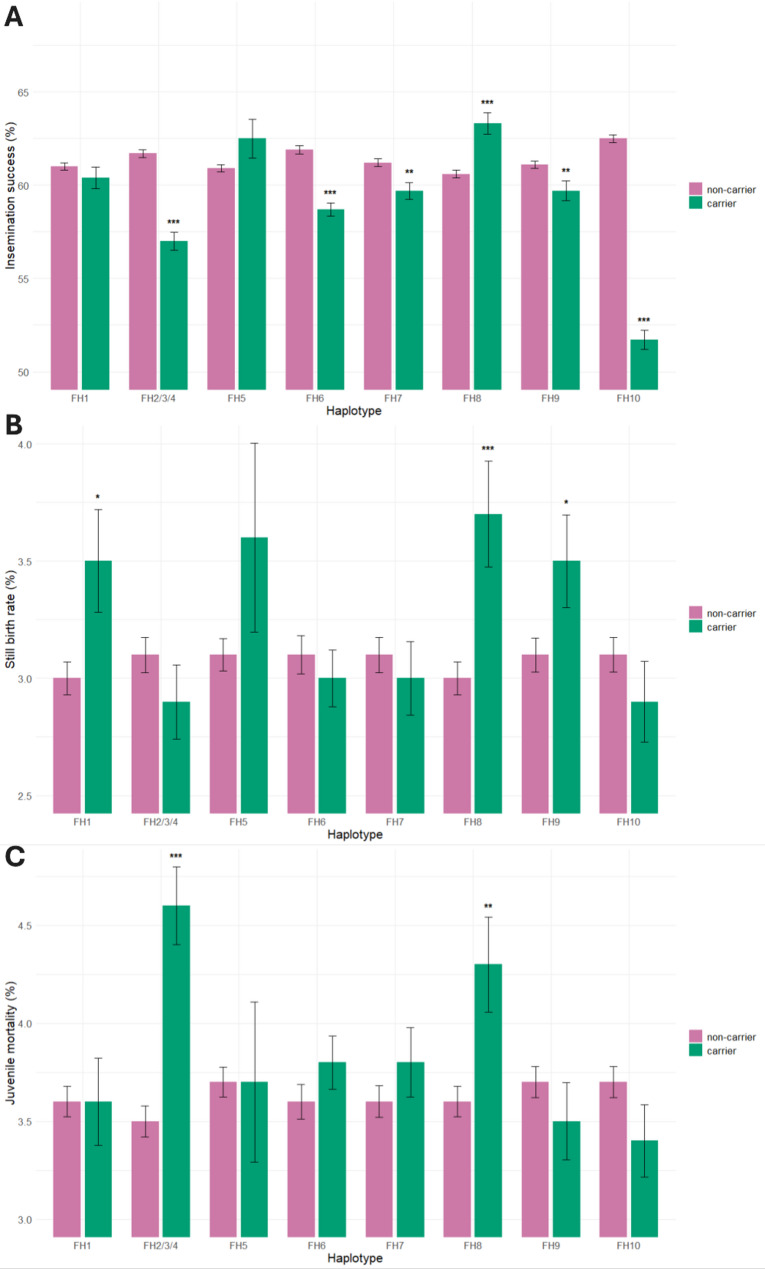
Insemination success, still birth rate and juvenile mortality of matings from non-carrier and carrier sires, for each haplotype. **A** Insemination success of matings from carrier sires and non-carrier sires. **B** Still birth rate of matings from carrier sires and non-carrier sires. **C** Juvenile mortality of matings from carrier sires and non-carrier sires. Non-carrier = all matings of non-carrier sires independent of the carrier status of the dam. Carrier = all matings of carrier sires independent of the carrier status of the dam. *P < 0.05, **P < 0.01, ***P < 0.001

The haplotypes FH1 and FH2/3/4 showed a significant relative reduction in insemination success in risk1 matings (carrier sire x carrier dam) compared to non-risk matings (non-carrier sire x carrier dam or carrier maternal grandsire) of 38.2% and 25.0%, respectively (Fig. 2; Additional file 2: Table S5). Moreover, FH1 also showed a significant increase in still birth rate in risk2 matings (carrier sire x carrier maternal grandsire) (Fig. 2; Additional file 2: Table S5). This could be explained by the FH1 region comprising the hydrocephalus mutation, which leads to still births or foals euthanized at birth to facilitate parturition [8]. When breeders do not register their foal as (still)born, the mother is marked as delivering no foal in that given year resulting in reduction in insemination success. In addition, FH8 also showed a significant increase in still birth rate in risk2 matings. This is likely a result of the FH8 region comprising the dwarfism mutation. Some foals born with dwarfism are directly euthanized, whereas others are euthanized at a later stage of life. Furthermore, FH7 showed a significant increase in juvenile mortality, but only in risk2 matings (Fig. 2; Additional file 2: Table S5). For the other haplotypes no clear phenotypic effects for both risk1 and risk2 matings compared to non-risk matings were observed. When comparing all matings of carrier sires to non-carrier sires, independent of the status of the dam or maternal grandsire, a significant reduction in insemination success of 2.3% to 17.3% (relative differences) was found for the FH2/3/4, for FH6, FH7, FH9, and FH10 (Fig. 3; Additional file 2: Table S6). In addition, a significant increase in still birth rate was found for FH1, FH8, FH9 (16.7 to 23.3%), and a significant increase in juvenile mortality was found for FH2/3/4 and FH8 (19.4% to 31.4%) (Fig. 3; Additional file 2: Table S6).

### Candidate causal variants identified

For each haplotype, two to 13 of the sequenced sires were carrier. Therefore, for each haplotype it was possible to identify variants that were exclusively shared by carriers of the haplotype and not present in non-carriers. Candidate causal variants were found for six of the ten haplotypes (Table 2). We were not able to determine a candidate causal variant for FH5, FH6, FH7, and FH9. Although there were some missense and splice region variants in high LD with FH9 (Additional file 2: Table S3), all of them were predicted tolerated by SIFT or were not conserved (Additional file 1: Fig. S2 and S3; Additional file 2: Table S7).

**Table 2:** Candidate causal variants for lethal haplotypes. Type of variant, location, reference allele, alternate allele, gene, amino acid (AA) change, relative position in the protein and the gene name for each of the lethal haplotypes. Positions are based on EquCab_Friesian_WUR (GCA_964023265.1). The asterisk indicates a premature stop codon.

Hap	Type	Chr	Position	Ref	Alt	Gene	AA change	Relative pos. in protein
FH1	Stop-gained	1	78,718,816	C	T	*B3GALNT2*	Q475*	Exon 12
FH2/3/4	Frameshift	4	76,214,165	CCACCATGAACACTGC	CC	*MET*	P423LQ*	Exon 1
FH8	Splice-region	14	9,618,724	C	T	*B4GALT7*		Exon 7
FH10	Deletion (261 Kb)	23	8,835,330–9,096,938	-	-	*ENSECAG00000060944*,* ENSECAG00000062694*,* ENSECAG00000072954*,* ENSECAG00000070952*,* ENSECAG00000075096*	-	-

#### The known causal mutations for dwarfism and hydrocephalus are identified

The haplotypes FH1 and FH8 comprise the causal mutations causing hydrocephalus and dwarfism, respectively. For hydrocephalus, four variants affecting the coding region (three missense, one stop gained) were in complete LD with FH1 (Additional file 2: Table S7). The stop-gained variant (1:78718816 C > T) has been identified as the causal mutation for hydrocephalus [8] (Table 2). For dwarfism, two variants affecting the coding region (missense) or splice region were in complete LD with FH8 (Additional file 2: Table S7). The missense variant was tolerated by SIFT (> 0.05) (Additional file 2: Table S7) and therefore not likely to be detrimental. The splice variant (14:9618724 C > T) in the Beta-1,4-galactosyltransferase 7 (*B4GALT7*) gene has been identified as the causal mutation for dwarfism [12] (Table 2). This causal variant affects a conserved cytosine and was found to have a high combined annotation dependent depletion (CADD) score of 35 in this conserved splice region between human and horse (Additional file 1: Fig. S4).

#### A 14-bp frameshift variant in proto-oncogene *MET* likely induces early lethality in Friesian horses (FH2/3/4)

**Fig. 4:**
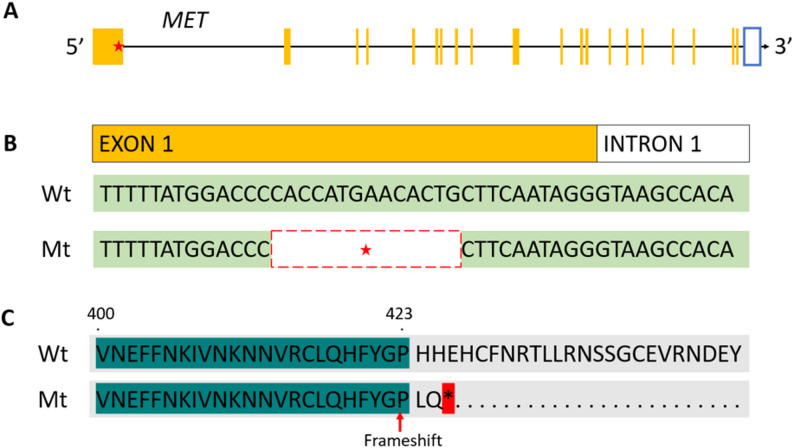
The frameshift (4:74041982CCACCATGAACACTGC > CC) variant in *MET* mapped against EquCab3.** A**. *MET* gene model. The location of the affected exon 1 is indicated with the red star. **B**. Illustration of the 14-bp deletion in exon 1 of *MET*. **C.** Alignment of the wild (Wt) and mutant type (Mt) *MET* protein sequence. The variant introduces 2 novel amino acids and a premature stop codon indicated with the red asterisk

The haplotypes FH2, FH3, and FH4 were in LD sharing partly the same carriers (Additional file 1: Fig. S1). FH2 and FH3 shared the same eight sequenced haplotype carriers, and FH4 only had four sequenced haplotype carriers of which three were shared by FH3 and FH2. Therefore, we first considered candidate causal variants shared by only the eight sequenced haplotype carriers of FH2 and FH3. We found three variants that affected the coding sequence (of which two missense and one splice region) and were in complete LD. One missense variant was predicted by SIFT to be tolerated and therefore unlikely to be the causal variant (Additional file 2: Table S7). The other missense variant in *EEPD1* had a SIFT score of 0.03, but one horse was found homozygous for that variant in the Ensembl variant database and therefore unlikely to be causal. Furthermore, the location of the splice variant (4:68325815 C > T) showed that some vertebrates already have a basepair T on that location (Additional file 1: Fig. S5), indicating that this variant is likely not deleterious. Notably, the Friesian horse that was used to develop the reference genome was included in the sequence data and was found to be a carrier of the haplotype. Therefore, we also checked for variants based on sequence data mapped against EquCab3. This resulted in two variants affecting the coding region (splice and frameshift) shared by all eight haplotype carriers plus one or two non-carriers of the haplotype (Additional file 2: Table S7). The splice region variant only affects one transcript and is therefore unlikely to be causal. However, the frameshift variant (4:74041982CCACCATGAACACTGC > CC) affecting the first exon of the proto-oncogene *MET*, is predicted to have a high impact. The frameshift introduces two novel amino acids followed by a premature stop codon in the first exon of *MET* (Fig. 4), causing a truncated protein. Visual inspection showed that the *MET* gene is broken when annotated in EquCab_Friesian_WUR (Additional file 1: Fig. S6), because the mutant haplotype is assembled in the reference genome. *MET* is a prototypical tyrosine kinase receptor and known as a proto-oncogene. *MET* supports a morphogenetic program, known as invasive growth, taking place both during embryogenesis and adulthood [39]. Moreover, homozygous *MET* null-mutant mice exhibit impaired placentas resulting in embryonic lethality [40, 41].

#### A 261-Kb deletion as likely causal variant affecting fertility rates (FH10)

Six sequenced sires were carrier of FH10, but no SNPs or SVs were in complete LD with FH10. When expanding the analysis to variants which were not in complete LD, we found two missense variants in the coding region and one large 261-Kb deletion encompassing ncRNAs which were shared by all six carrier sires plus two or three non-carriers of the haplotype (Additional file 2: Table S7). All three non-carriers were heterozygous for different low frequency haplotypes (< 0.1%) which were very similar to the lethal haplotype, making them likely to also carry the candidate causal variant. Both missense variants are tolerated by SIFT (Additional file 2: Table S7), and therefore unlikely to be the causal variant. Therefore, the 261-Kb deletion (Additional file 1: Fig. S7), given its large size and some ncRNAs being located within it, was considered to be the likely candidate for the causal variant (Table 2).

To get a better understanding of the function of the ncRNAs involved, we used the nucleotide BLAST algorithm (NCBI, discontiguous Mega BLAST) to study complementary RNA sequences in *Homo sapiens*. The ncRNAs were found to overlap with the spermatogenesis associated 31 subfamily D (*SPATA31D*) genes (Additional file 2: Table S8). In addition, when mapped against EquCab3 (GCA_002863925.1) and TB-T2T (GCA_041296265.1), the deletion was 356-kb and 346-kb respectively and comprised solute carrier family 28 member 3-like (*SLC28A3*) and spermatogenesis-associated protein 31D like genes (*SPATA31D*) (Additional file 1: Fig. S8 and S9). The *SPATA31D* genes are uniquely expressed in the mammalian testes [42]. To further validate whether *SPATA31D* genes are located in the deleted region, we mapped available RNA sequence data from three immature and three mature testes of Mongolian sires against EquCab_Friesian_WUR. This showed clear RNA expression in the 261-Kb deleted region, with increased expression in mature testes compared to immature testes (Additional file 1: Fig. S10). In addition, FPKM and TPM values for the 261-kb deleted region were ~ 5 fold higher for mature testes compared to immature testes (Additional file 2: Table S9). Therefore, we also tested if there was a difference between semen quality parameters of carriers and non-carriers of the 261-kb deletion. Interestingly, carriers (variant found in sequence data or haplotype carrier based on genotype data) had a significantly higher percentage of living sperm cells with abnormal head shape compared to non-carriers (Additional file 2: Table S10).

#### PCR validation of the 14-bp deletion on FH2/3/4 and the 261-kb deletion on FH10

For the haplotypes comprising the 14-bp deletion and 261-kb deletion, we observed a few homozygous animals based on 70 K SNP data (Table 1). We hypothesized that those animals have undergone recombination between the haplotype and the candidate lethal variant, causing them to be homozygous for the lethal haplotype, but heterozygous for the candidate lethal variant. PCR results showed that the animals homozygous for the haplotype FH3 and FH10 were indeed heterozygous for the candidate variant (Additional file 1: Figs. S11 and S12; Additional file 2: Table S11), thereby providing strong evidence that the candidate variants for those haplotypes are causal and likely result in lethality in homozygous state.

## Discussion

In this study, we scanned for missing homozygous haplotypes in 8,263 horses and reported 10 likely harmful haplotypes with carrier frequencies of 8.0% to 22.1% in the Friesian horse breed. We were able to pinpoint candidate causal variants for six haplotypes, using sequence data of 50 influential sires. Two haplotypes comprised the known causal variants for hydrocephalus [8] and dwarfism [12], respectively. This supports the reliability of our approach. For both haplotypes no homozygous animals were found, which was expected given that breeders prevent carrier-by-carrier matings using the available DNA tests for dwarfism and hydrocephalus. For four haplotypes, of which three were in LD, we identified two novel candidate variants likely causing lethality in homozygous state. For those haplotypes, there were a few homozygous animals, possibly suggesting incomplete linkage disequilibrium of the haplotypes with the causal variant. PCR validation showed that the animals homozygous for the haplotype were indeed heterozygous for the candidate variant, likely due to recombination. Thereby providing strong evidence that those candidate variants cause lethality in homozygous state and illustrating that the carrier status based on haplotype is not fully reliable to identify carrier animals. Thus, a DNA test should be developed for those novel likely causal variants to identify carrier animals and thereby preventing risk matings. For some haplotypes, we did not find candidate causal variants, which could be due to causal variants outside the coding region which are more difficult to fine-map. Here, only the most significant haplotypes (P adjusted < 0.05) were published. With the continuous routine collection of genotypes, the power to detect haplotypes will further increase in coming years, allowing the detection of haplotypes with even lower frequencies or those with even smaller effects on fertility and/or mortality.

We identified a 14-bp deletion in the *MET* proto-oncogene as the candidate causal variant for FH2/3/4. This gene encodes a transmembrane tyrosine kinase identified as the receptor of a polypeptide known as the hepatocyte growth factor. Several mutations in *MET* were found to be associated with severe disorders in humans, like deafness [43], renal cell carcinoma [44], muscular dysplasia and arthrogryposis [45], osteofibrous dysplasia [46], and childhood hepatocellular carcinoma [47]. In addition, mice homozygous for a mutation in *MET* were embryonic lethal and had complete loss of muscles that originated from migratory precursors. Heterozygous mice were also affected; they were born alive but showed reduction in the number of myofibers in both appendicular and axial muscles [45]. Also, homozygous *MET* null-mutants exhibit impaired placentas resulting in embryonic lethality [40, 41]. In the Friesian horse population, the theoretically expected 25% decrease in insemination success was found in risk1 matings. However, we could only detect a decrease in insemination success and, therefore, the stage of the pregnancy at which the lethality occurs in Friesian horses should be further investigated. Still, based on the phenotypic effect in homozygous mutant mice, we predict that this candidate causal variant is likely to result in embryonic lethality. Furthermore, a significant increase in juvenile mortality was found between matings of carrier sires compared to matings of non-carrier sires. However, this was not significant in risk1 and risk2 matings compared to non-risk matings. Therefore, the observed increase in juvenile mortality amongst offspring of carrier sires may be influenced by factors other than the candidate causal variant. Nevertheless, a potential effect of the variant on juvenile mortality cannot be excluded, and more observations are needed to draw any conclusions. In the near future, a genetic test could be developed which may be useful for breeders to prevent risk-matings and thereby increase the insemination success.

A large 261-kb deletion was identified as the candidate causal variant for FH10. No significant difference between risk and non-risk matings was found, which could be explained by the small number of risk matings. Interestingly, there was a much larger than expected significant reduction (17.3%, compared to the expected 4.1% reduction (0.5 * 0.5 * 16.5% carrier frequency) in insemination success for all matings of carrier sires, independent of the carrier status of the dam (Fig. 3; Additional file 2: Table S6). This could indicate that the candidate causal variant affects fertility of carrier sires, independent of the dam’s carrier status. This hypothesis is strengthened by the ncRNAs found in the deleted region which are complementary with the testis-specific *SPATA31D* gene family in humans, involved in spermatogenesis. While the exact function of *SPATA31D* remains unknown, several genes from the *SPATA* family have been shown to be associated with sperm head formation. For example, *SPATA1* was found to be a strong candidate for the shape of the sperm head [48], mutations in *SPATA16* cause absence of the acrosome [49], and mutations in *SPATA20* lead to headless spermatozoa [49]. Notably, we found a small significant increase in the number of alive sperm cells with abnormal head shape in carrier sires. However, only data from approved sires was available, which is biased as those sires have to meet certain semen quality levels in order to get their approved status. Therefore, in a few years, it would be valuable to analyze the semen quality of all genotyped sires born from 2023 onwards. Furthermore, RNA-seq data of the testis confirmed expression in the deleted region, where mature testis had higher expression compared to immature testes (Additional file 1: Fig. S10), suggesting that this region is involved in maturation processes of the testis. Ing et al. (2020), found greater expression levels of *SPATA31D* genes in denser spermatozoa (superior motility, morphology and chromatin structure) resulting in greater pregnancy rates compared to less dense sire spermatozoa [42]. Moreover, homozygous KO mice for *SPATA31* genes showed male infertility [50]. Thus, it is possible that the 261-kb deletion affects spermatogenesis and thereby reduces insemination success. Still, the specific pathway of this regulation is not yet clear and needs further investigation.

The candidate variant for FH10 is expected to not only reduce semen quality but is also to cause lethality in homozygous state as there was an absence of homozygous animals for the variant (Additional file 1: Fig. S12; Additional file 2: Table S11). It is believed that long ncRNAs in spermatozoa regulate gene expression during early embryonic development [42]. Therefore, the loss of long ncRNAs resulting from the candidate deletion may impair embryonic development, potentially explaining the absence of homozygous individuals carrying the candidate variant. However, in that case we would expect a clear significant reduction in insemination success, still birth rate or juvenile mortality between risk and non-risk matings. As this was not observed, it could be that the sire spermatozoa which are affected do not fertilize, but the dam is still fertilized by spermatozoa carrying no lethal variant. This could explain the absence of homozygous haplotypes. Still, this does not explain why we do observe a larger than expected significant reduction in insemination success between carrier sires and non-carrier sires. Therefore, the most likely explanation is that this haplotype affects both semen quality and causes lethality in homozygous state, but due to the relatively low number of risk matings we do not observe a phenotypic difference. In addition, it is known that the mating registration is not complete, possibly causing no clear phenotypic effect for this haplotype.

Only for FH1 and FH2/3/4 we found a significant reduction in insemination success in risk1 matings. The reason why we found no clear phenotypic differences in risk1 matings for the other haplotypes could be due to the relatively low number of available risk1 matings (between 16 and 203). It is also known that mating registration is incomplete. For example, for a dam that needs three inseminations in different cycles to get pregnant, the sire keeper will likely only register the last successful insemination. Thus, based on mating data, it seems like the dam got pregnant after one insemination, while in reality more attempts were required. It is not known how frequent only the successful mating is registered but insemination success will consequently be biased upward, especially for risk1 matings where the number of matings is relatively low and thereby more influenced by incomplete registrations. Thus, this illustrates the importance of complete mating registration to validate the phenotypic effect of causal variants.

The approach in our study does not allow detecting all embryonic lethal variants present in the current population. Recent variants likely go by undetected, as only a small fraction of the horses with the specific haplotype would carry the recent variant. Thus, our approach focused on detection of variants which are segregating for a long time in the population and thereby got the chance to reach moderate to high frequencies. This is also reflected by the most recent common ancestors of the sequenced carriers of FH1, FH2/3/4, FH8, and FH10, which were born between 1967 and 1986. While the causal mutation for dwarfism has only been reported in Friesian horses [12], the causal mutation for hydrocephalus has also been incidentally found in Belgian draft and Warmblood horses [51, 52]. Whether the candidate causal variants for FH2/3/4 and FH10 are breed-specific should be further investigated. Notably, the potential lethal haplotype on chromosome 6 found in thoroughbreds [20] did not appear in our analysis. This could indicate that the causal variant is not present in the Friesian horse breed or only present as a rare variant and therefore not significantly detected in this analysis. Furthermore, we predicted missense variants as likely deleterious based on SIFT scores [29]. However, additional predictions from tools such as PolyPhen [53] or PROVEAN [54] could be incorporated in future analyses to further investigate such variants.

Carrier frequencies of the six harmful haplotypes for which we identified candidate causal variants ranged from 8.0 to 16.5% in the Friesian horse population. While lethal alleles can reach up to 20% carrier frequency by genetic drift alone [7], it is also possible that these frequencies of lethal haplotypes are driven by heterozygote advantage for favorable breeding goal traits. This has already been shown in several livestock species [55]. For example, a recessive mutation causing embryonic lethality reached carrier frequencies of 13, 23, and 32% in Danish, Swedish, and Finnish red cattle, respectively, due to its positive effects on milk yield and composition in carriers [56]. However, the current genotype dataset consists only of a few horses which also had phenotypic records for breeding goal traits available. Therefore, the current dataset will not provide enough power to detect any heterozygote advantage. The increasing amount of genotype data in the coming years as well as DNA tests that could be developed, would make it possible to investigate heterozygote advantage in the future.

Here, we used the EquCab_Friesian_WUR to analyze haplotype regions and to identify variants in sequence data. The use of a breed-specific reference genome removes between breed variation and thus increases the likelihood of pinpointing the likely causal variant. However, as demonstrated for FH2/3/4, a drawback is that the variant may also be present in the reference genome itself, making it more difficult to identify the likely causal variant. As a solution, we also mapped sequence data to EquCab3, allowing us to detect the candidate causal variant. This candidate causal variant for FH2/3/4 was also detected in sequence data mapped to EquCab_Friesian_WUR, but with an allele frequency of 54%, and 47 of the 50 sequenced sires were found to be carrier. Visual inspection showed that the genotypes of this variant were not correctly called against EquCab_Friesian_WUR, and therefore identification of the candidate causal variant relied on EquCab3.

In the near future, it would be valuable to develop DNA tests for those two novel candidate causal variants to prevent carrier-by-carrier matings. Still, selection would be necessary to reduce mutation frequencies. Selection against all causal variants simultaneously will be highly unrealistic, as there are multiple causal variants segregating at moderate frequencies in the Friesian horse. In such situations, there are few animals that are free of all undesired alleles. If only those animals were used for breeding, genetic diversity would be severely compromised and other disorders might appear over time. Therefore, it could be considered that the strength of selection depends on the severity caused by each variant. For example, the mutations causing dwarfism and hydrocephalus might be of higher severity than the two novel likely lethal recessive variants. Namely, while the novel likely lethal recessive variants reduce insemination success, the mutations causing dwarfism and hydrocephalus allow a full gestation period, but result in a genetically deviated foal, thereby both hampering the pregnancy success leading to a healthy foal as well as raising welfare concerns for the produced offspring [8, 12]. Adding to that, hydrocephalus is also associated with dystocia and could even lead to fatal complications for the dam at parturition [8]. Moreover, when in the future multiple less frequent lethal variants are discovered, those could be combined in a breeding value, thereby still selecting against the lower frequent variants, and maintaining the ease for breeders to prevent carrier-by-carrier matings for the moderate to high frequent lethal variants.

## Conclusions

Our study is one of the first in its kind to analyze homozygous deficient haplotypes and report candidate lethal variants in horses. Here, we show that large scale genotype data in combination with phenotype and whole genome sequence data provides a powerful resource to identify candidate causal variants affecting pre- or postnatal survival in Friesian horses. We report 10 likely harmful haplotypes with carrier frequencies between 8.0% and 22.1% in the Friesian horse breed. We identified two novel candidate variants affecting fertility rates in Friesian horses. DNA tests for those variants could be developed in the near future, thereby indirectly improving insemination success in the Friesian horse. Considering the population characteristics of domestic horse breeds, we strongly recommend performing such analyses in other horse breeds using the increasingly available genotype data. These studies would provide an important contribution to the improvement of fertility rates in horse populations.

## Supplementary Information

Additional file 1: FigS1. Venn diagram of the carriers of FH2. FH3 and FH4. Venn diagram created using the online webtool https://bioinformatics.psb.ugent.be/webtools/Venn/. FigS2. The splice variant (18:18323952G>A) in MGAT5. A. The splice variant in one of the carrier animals mapped against EquCab3 and visualized in JBrowse. B. Screenshot of UCSC Genome Browser on Human. The location of the splice variant in other vertebrates shows that also other vertebrates visualized already have a basepair A on that location (marked yellow). indicating that this variant is likely not deleterious. The splice variant has a combined annotation dependent depletion (CADD) score of 6.2 (A>G). FigS3. The splice variant (18: 18936171TG>CA) in RAB3GAP1. A. The splice variant in one of the carrier animals mapped against EquCab3 and visualized in JBrowse. The variant of the carrier animal mapped against EquCab is 18:19132248CA>TG. B. Screenshot of UCSC Genome Browser on Human. The location of the splice variant in other vertebrates shows that most vertebrates visualized already have a basepair T and G on that location (marked yellow). indicating that this variant is likely not deleterious. The splice variant has a combined annotation dependent depletion (CADD) score of 13.4 (T>C) and 6.2 (G>A). FigS4. The splice variant (14:9618724C>T) in B4GALT7. A. The splice variant (14:9618724C>T) in one of the carrier animals mapped against EquCab3 and visualized in JBrowse. B. Screenshot of UCSC Genome Browser on Human. The location of the splice variant is conserved and the variant to T has a combined annotation dependent depletion (CADD) score of 35. FigS5. The splice variant (4: 68325815C>T) in ALNL. A. The splice variant (4:68325815C>T) in one of the carrier animals mapped against EquCab3 and visualized in JBrowse. B. Screenshot of UCSC Genome Browser on Human. The location of the splice variant in other vertebrates shows that most vertebrates visualized already have a basepair T on that location (marked yellow). indicating that this variant is likely not deleterious. The splice variant has a combined annotation dependent depletion (CADD) score of 6.0 (T>C). FigS6. Visualisation of the frameshift variant (4:76214165CCACCATGAACACTGC>CC) on chromosome 4 in JBrowse. A. Visualisation of the frameshift variant on EquCab3 reference genome. The grey area with a star indicates the small deletion causing a frameshift. B. Visualisation of the frameshift variant on the EquCab_Friesian_WUR reference genome. The purple I indicates an insertion and carriers of the frameshift variant do have ~50% normal reads (without insertion) as the reference genome horse is also carrier. Ensembl annotation shows that one transcript is broken caused by the small deletion in the reference genome horse. FigS7. Visualization of the 261-Kb deletion on chromosome 23 in JBrowse against EquCab_Friesian_WUR. The red arrow indicates the decrease in coverage in the carrier animal caused by the deletion. Ensembl annotation is shown and five ncRNAs are (partly) affected by the deletion. FigS8. Visualization of the 356-Kb deletion on chromosome 23 in JBrowse against EquCab3. The red arrow indicates the decrease in coverage in the carrier animal caused by the deletion. Ensembl annotation is shown. FigS9. Visualization of the 346-Kb deletion on chromosome 23 in JBrowse against T2T assembly. The red arrow indicates the decrease in coverage in the carrier animal caused by the deletion. NCBI annotation is shown. FigS10. JBrowse screenshot of the RNA sequence data mapped against EquCab_Friesian_WUR in a part of the region comprising the 261-Kb deletion. Both RNA expression in immature and mature testis are shown. FigS11. Results of the fragment analysis on ABI3730 for the 14-bp deletion on chromosome 4. The fragment analysis shows that F1-4 are homozygous wildtype and F5-6 are heterozygous for the 14-bp deletion. FigS12. Results of the agarose gel for testing on the 262-kb deletion. The results show that F1-4 are heterozygous for the 261—kb deletion and F5-6 are homozygous wildtype. FigS13. Quality of gDNA for the 6 samples for PCR validation was checked on 0.8% agarose gel, 40 volt and for three hours. All six samples show more than sufficient gDNA quality.

Additional file 2: Table S1. The filtered 40,809 SNPs that were included in the analysis. The table shows .bim file including the chromosome, SNP id, position, allele1 and allele 2. Table S2. Quality of gDNA for the six samples for PCR validation was checked on Denovix and Qubit. Quality of the gDNA of all six animal samples (F1 – F6) was sufficient. Table S3. Primer design for PCR validation of two candidate variants. Primer design for the 14-bp frameshift deletion on chromosome 4 and the 261-kb deletion on chromosome 23. Table S4. Haplotypes with homozygous deficiency including the haplotype sequences. The 10 haplotypes with homozygous deficiency based on EquCab_Friesian_WUR reference genome with Bonferroni adjusted P < 0.05. In total, 70K SNP Chip data of 8,263 horses were included in the analysis. The table shows the genomic location and the haplotype sequences. The SNP IDs and their location of each SNP in the haplotype sequence can be found in Table S1. Table S5. The number of matings, insemination success, still birth rate and juvenile mortality in risk1, risk2 and non-risk matings. The relative differences in % for insemination success, still births and juvenile mortality of risk matings compared to non-risk matings are represented. To illustrate, for FH1, non-risk matings had an average insemination success of 76.4%. Risk1 matings had 38.2% lower insemination success compared to non-risk matings, resulting in average insemination success of 47.2% (76.4% – (76.4%*0.382)). Risk1 matings were defined when both the sire and dam were carrier. Risk2 matings were defined when the sire was carrier and the maternal grandsire was carrier. Non-risk matings were defined when the sire was non-carrier and the dam was carrier or maternal grandsire was carrier. Table S6. The number of matings, insemination success, still birth rate and juvenile mortality in matings of carrier sires and non-carrier sires. The relative differences in % for insemination success, still births and juvenile mortality of carrier sires compared to non-carrier sires are represented. To illustrate, for FH1, matings of non-carrier sires had an average insemination success of 61.0%. Matings of carrier sires had 1.0% lower insemination success compared to non-risk matings, resulting in average insemination success of 60.4% (61.0% – (61.0%*0.01)). All matings of carrier sires and non-carrier sires were included, independent of the carrier status of the dam or maternal grandsire. Table S7. High impact variants per candidate lethal haplotype. All high impact variants found per candidate lethal haplotype. Table S8. nBLAST results of ncRNAs in the 261-kb deletion of FH10. Discontiguous megablast was performed to look for similar RNA sequences in Homo sapiens. All similar RNA sequences with an E value < 10-35 are reported. Table S9. FPKM (fragments per kilobase per million fragments mapped) and TPM (transcripts per kilobase million) values for immatures testes and mature testes of six Mongolian horses. Table S10. Semen quality parameters between non-carriers and carriers of FH10. Median and interquartile range (IQR) are calculated for both non-carriers and carriers of FH10. *P*-value using the Wilcoxon rank sum test was calculated to test difference in median for semen quality parameters between non-carriers and carriers of the 261-kb deletion. P_adj is the Bonferroni-adjusted *P*-value. Table S11. PCR results of two tested candidate variants in six horses. F1 – F4 animals are homozygous wildtype for the 14-bp frameshift deletion and heterozygous for the 261-kb deletion. F5 and F6 animals are heterozygous for the 14-bp frameshift deletion and homozygous wildtype for the 261-kb deletion.

Additional file 3.

## Data Availability

The WGS variants (SNPs and SVs) in and surrounding the lethal haplotypes (+/- 3Mb) in this study have been deposited in the European Variant Archive ( https://www.ebi.ac.uk/eva/) with the primary accession code ERP175022. The 70K genotypes of the candidate haplotype regions (+/- 3Mb) are provided in the supplementary information (Additional file 3).
