# Supplementary material for: Deficiency in homozygous haplotypes reveals recessive lethal variants affecting fertility and viability in the Friesian horse
Source: BMC Genomics. 2026 Mar 11;27:389. doi: 10.1186/s12864-026-12728-5 (PMC13088515; doi:10.1186/s12864-026-12728-5)
Supplement: Supplementary file 1 — Additional file 1: FigS1. Venn diagram of the carriers of FH2. FH3 and FH4. Venn diagram created using the online webtool https://bioinformatics.psb.ugent.be/webtools/Venn/. FigS2. The splice variant (18:18323952G>A) in MGAT5. A. The splice variant in one of the carrier animals mapped against EquCab3 and visualized in JBrowse. B. Screenshot of UCSC Genome Browser on Human. The location of the splice variant in other vertebrates shows that also other vertebrates visualized already have a basepair A on that location (marked yellow). indicating that this variant is likely not deleterious. The splice variant has a combined annotation dependent depletion (CADD) score of 6.2 (A>G). FigS3. The splice variant (18: 18936171TG>CA) in RAB3GAP1. A. The splice variant in one of the carrier animals mapped against EquCab3 and visualized in JBrowse. The variant of the carrier animal mapped against EquCab is 18:19132248CA>TG. B. Screenshot of UCSC Genome Browser on Human. The location of the splice variant in other vertebrates shows that most vertebrates visualized already have a basepair T and G on that location (marked yellow). indicating that this variant is likely not deleterious. The splice variant has a combined annotation dependent depletion (CADD) score of 13.4 (T>C) and 6.2 (G>A). FigS4. The splice variant (14:9618724C>T) in B4GALT7. A. The splice variant (14:9618724C>T) in one of the carrier animals mapped against EquCab3 and visualized in JBrowse. B. Screenshot of UCSC Genome Browser on Human. The location of the splice variant is conserved and the variant to T has a combined annotation dependent depletion (CADD) score of 35. FigS5. The splice variant (4: 68325815C>T) in ALNL. A. The splice variant (4:68325815C>T) in one of the carrier animals mapped against EquCab3 and visualized in JBrowse. B. Screenshot of UCSC Genome Browser on Human. The location of the splice variant in other vertebrates shows that most vertebrates visualized already have a basepair T on that locati [file 12864_2026_12728_MOESM1_ESM.docx]

**Supplemental figures.**


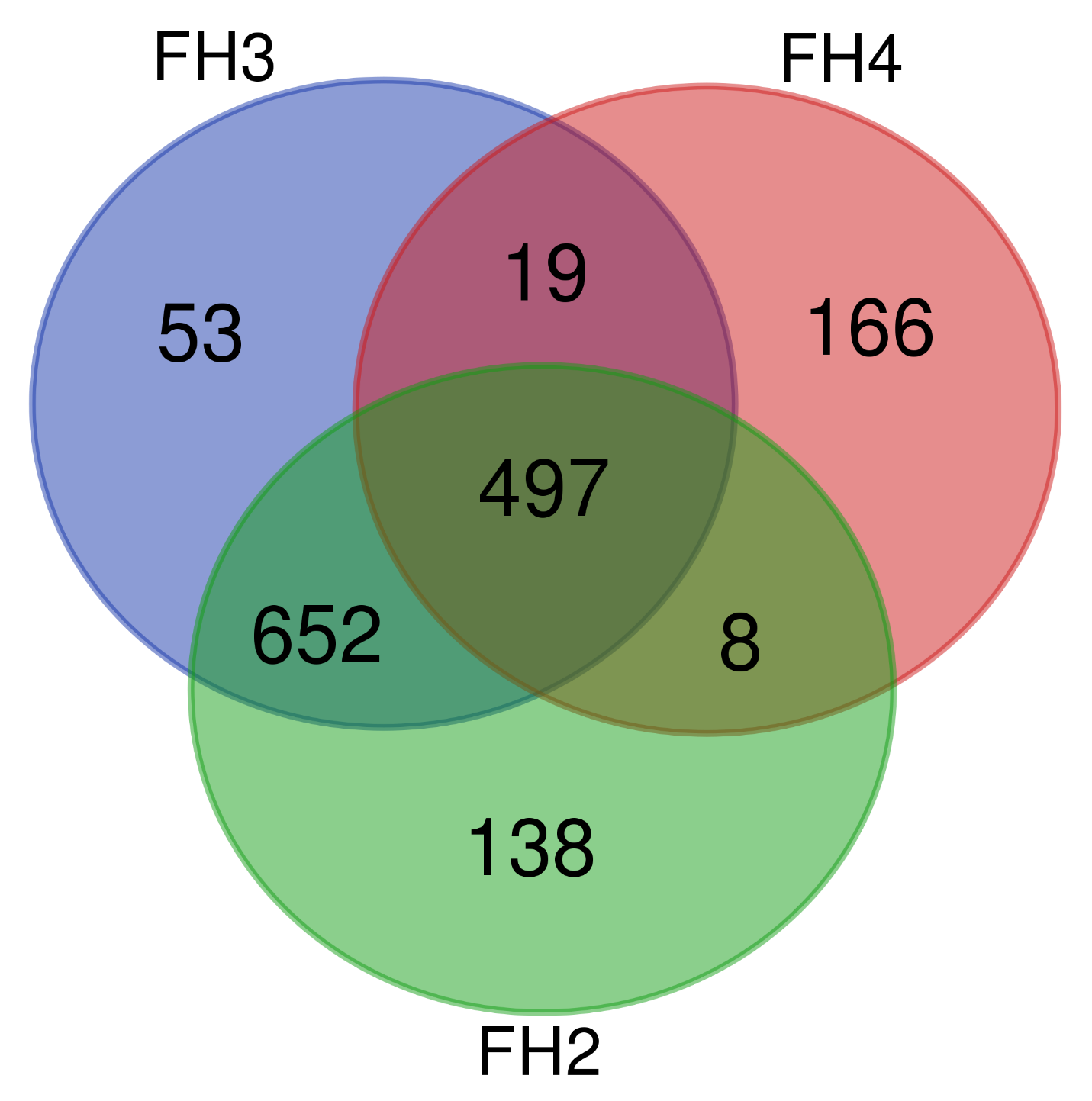


**Figure S1. Venn diagram of the carriers of FH2. FH3 and FH4.** Venn diagram created using the online webtool <https://bioinformatics.psb.ugent.be/webtools/Venn/>.

**
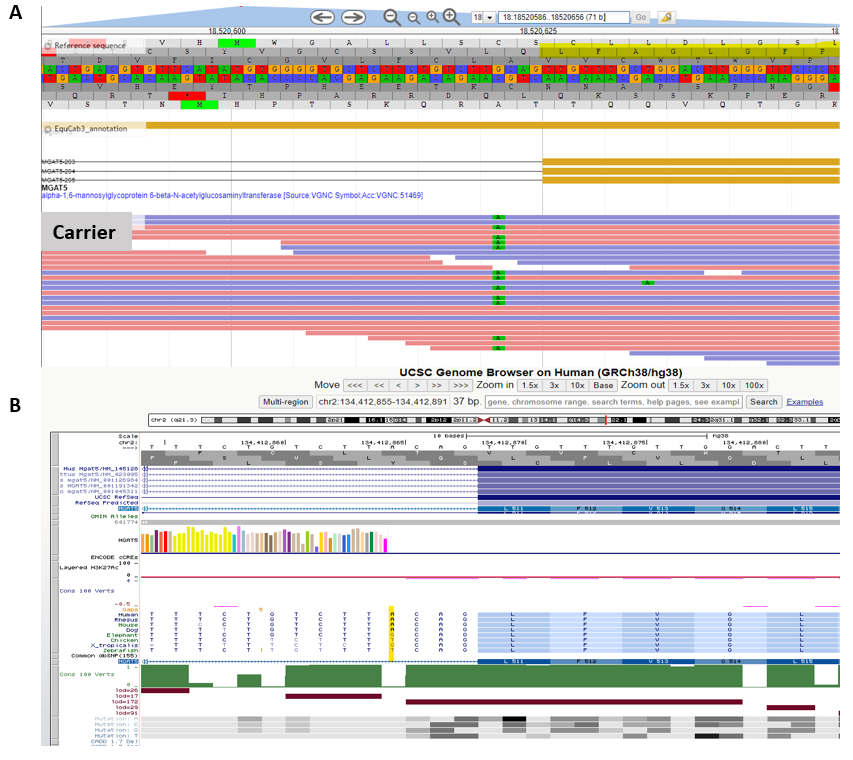
**

**Figure S2. The splice variant (18:18323952G>A) in *MGAT5*. A.** The splice variant in one of the carrier animals mapped against EquCab3 and visualized in JBrowse. **B.** Screenshot of UCSC Genome Browser on Human. The location of the splice variant in other vertebrates shows that also other vertebrates visualized already have a basepair A on that location (marked yellow). indicating that this variant is likely not deleterious. The splice variant has a combined annotation dependent depletion (CADD) score of 6.2 (A>G).

**
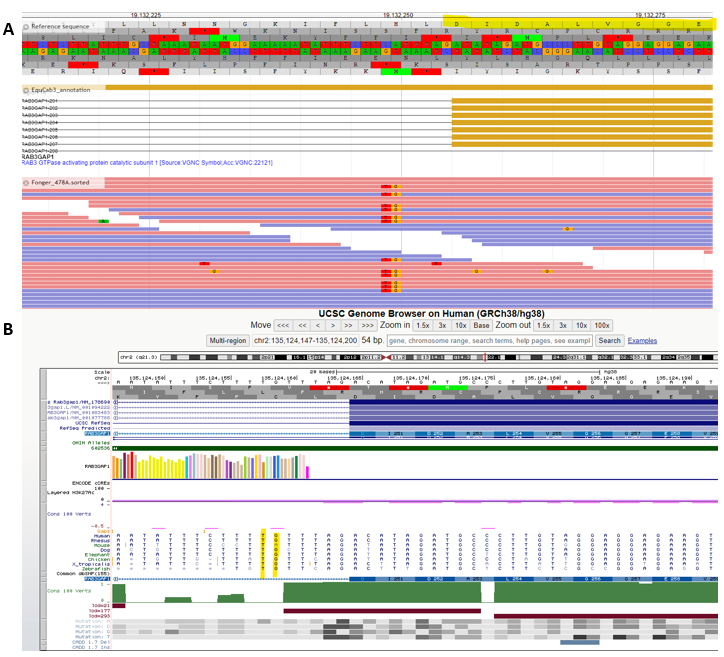
**

**Figure S3. The splice variant (18: 18936171TG>CA) in *RAB3GAP1*. A.** The splice variant in one of the carrier animals mapped against EquCab3 and visualized in JBrowse. The variant of the carrier animal mapped against EquCab is 18:19132248CA>TG. **B.** Screenshot of UCSC Genome Browser on Human. The location of the splice variant in other vertebrates shows that most vertebrates visualized already have a basepair T and G on that location (marked yellow). indicating that this variant is likely not deleterious. The splice variant has a combined annotation dependent depletion (CADD) score of 13.4 (T>C) and 6.2 (G>A).


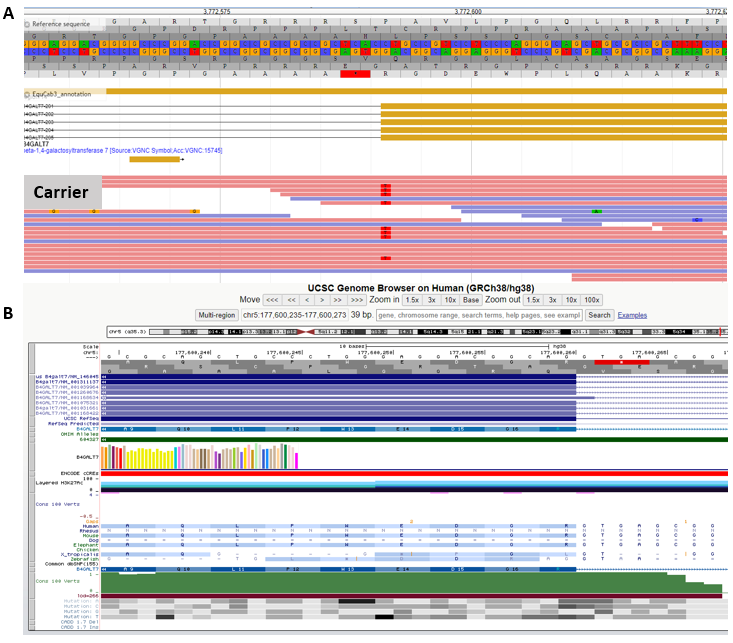


**Figure S4. The splice variant (14:9618724C>T) in *B4GALT7*. A.** The splice variant (14:9618724C>T) in one of the carrier animals mapped against EquCab3 and visualized in JBrowse. **B.** Screenshot of UCSC Genome Browser on Human. The location of the splice variant is conserved and the variant to T has a combined annotation dependent depletion (CADD) score of 35.


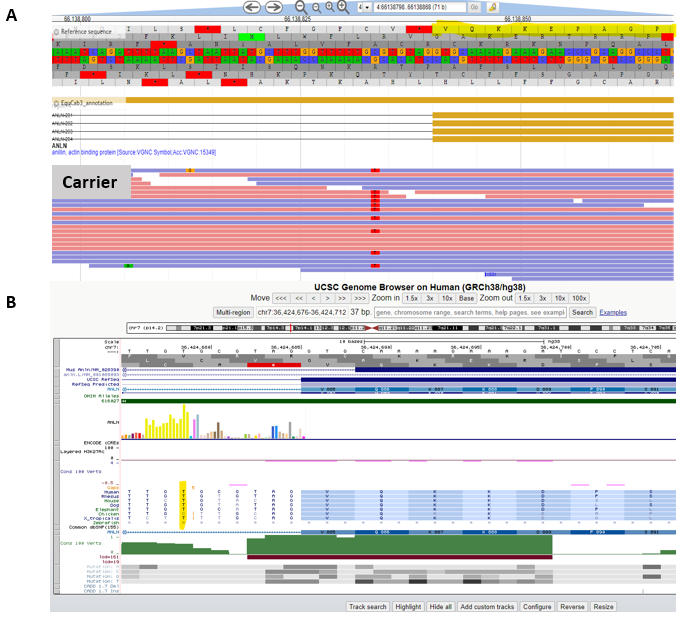


**Figure S5. The splice variant (4: 68325815C>T) in *ALNL*. A.** The splice variant (4:68325815C>T) in one of the carrier animals mapped against EquCab3 and visualized in JBrowse. **B.** Screenshot of UCSC Genome Browser on Human. The location of the splice variant in other vertebrates shows that most vertebrates visualized already have a basepair T on that location (marked yellow). indicating that this variant is likely not deleterious. The splice variant has a combined annotation dependent depletion (CADD) score of 6.0 (T>C).


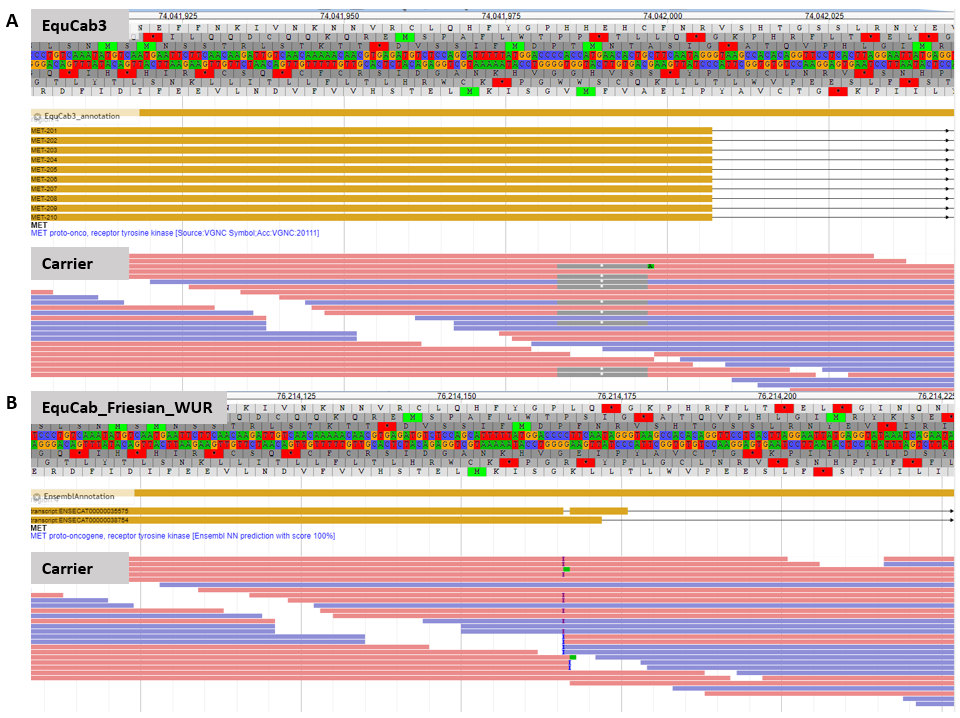


**Figure S6. Visualisation of the frameshift variant (4:76214165CCACCATGAACACTGC>CC) on chromosome 4 in JBrowse**. **A.** Visualisation of the frameshift variant on EquCab3 reference genome. The grey area with a star indicates the small deletion causing a frameshift. **B.** Visualisation of the frameshift variant on the EquCab_Friesian_WUR reference genome. The purple I indicates an insertion and carriers of the frameshift variant do have ~50% normal reads (without insertion) as the reference genome horse is also carrier. Ensembl annotation shows that one transcript is broken caused by the small deletion in the reference genome horse.


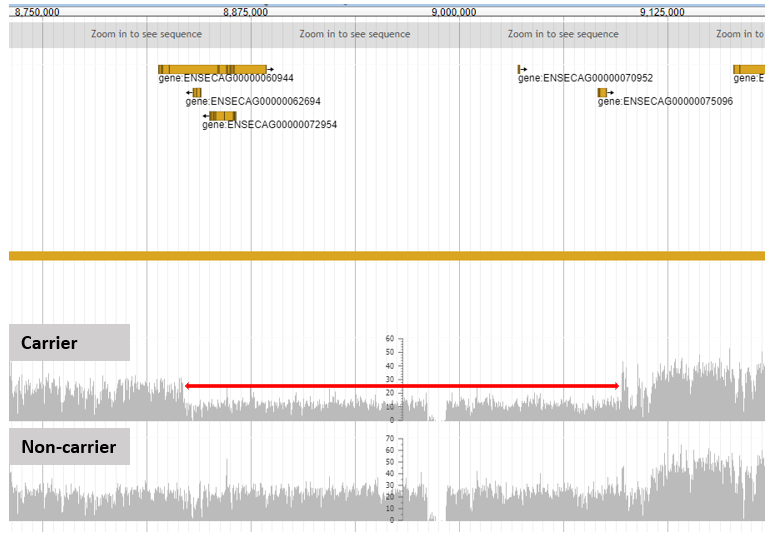


**Figure S7. Visualization of the 261-Kb deletion on chromosome 23 in JBrowse against EquCab_Friesian_WUR.** The red arrow indicates the decrease in coverage in the carrier animal caused by the deletion. Ensembl annotation is shown and five ncRNAs are (partly) affected by the deletion.

**
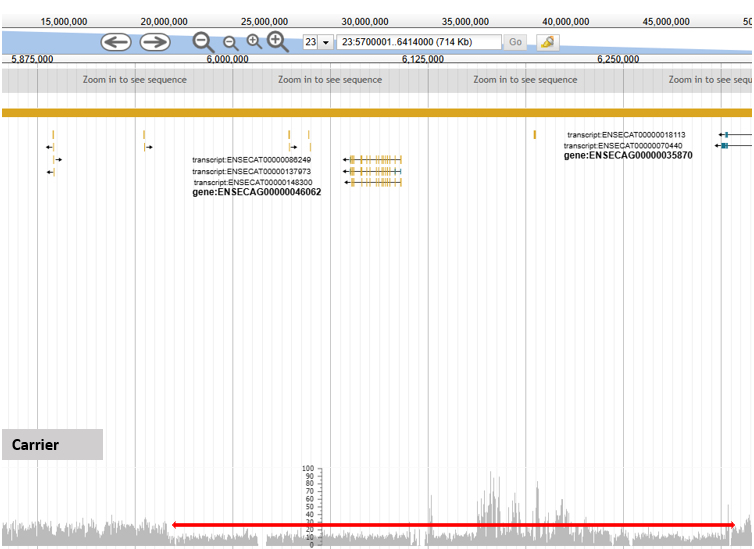
**

**Figure S8. Visualization of the 356-Kb deletion on chromosome 23 in JBrowse against EquCab3.** The red arrow indicates the decrease in coverage in the carrier animal caused by the deletion. Ensembl annotation is shown.


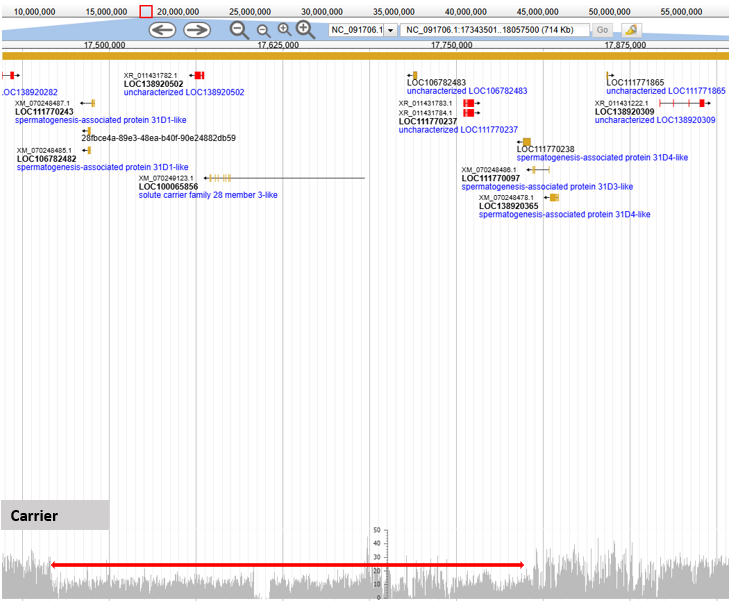


**Figure S9. Visualization of the 346-Kb deletion on chromosome 23 in JBrowse against T2T assembly.** The red arrow indicates the decrease in coverage in the carrier animal caused by the deletion. NCBI annotation is shown.


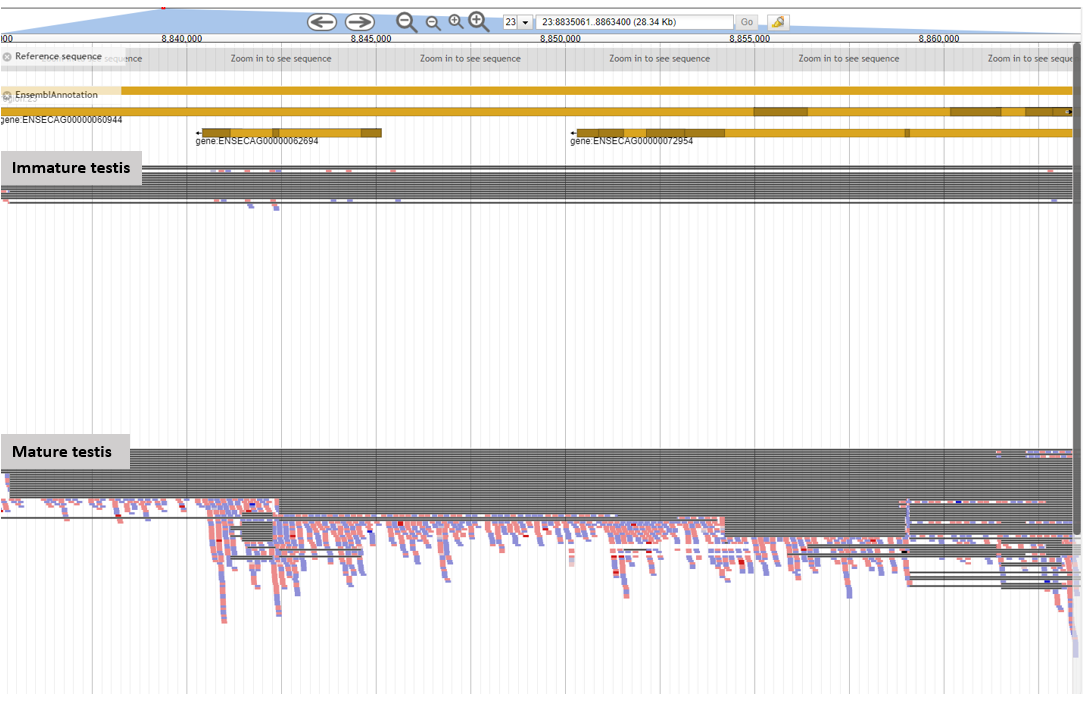


**Figure S10. JBrowse screenshot of the RNA sequence data mapped against EquCab_Friesian_WUR in a part of the region comprising the 261-Kb deletion.** Both RNA expression in immature and mature testis are shown.


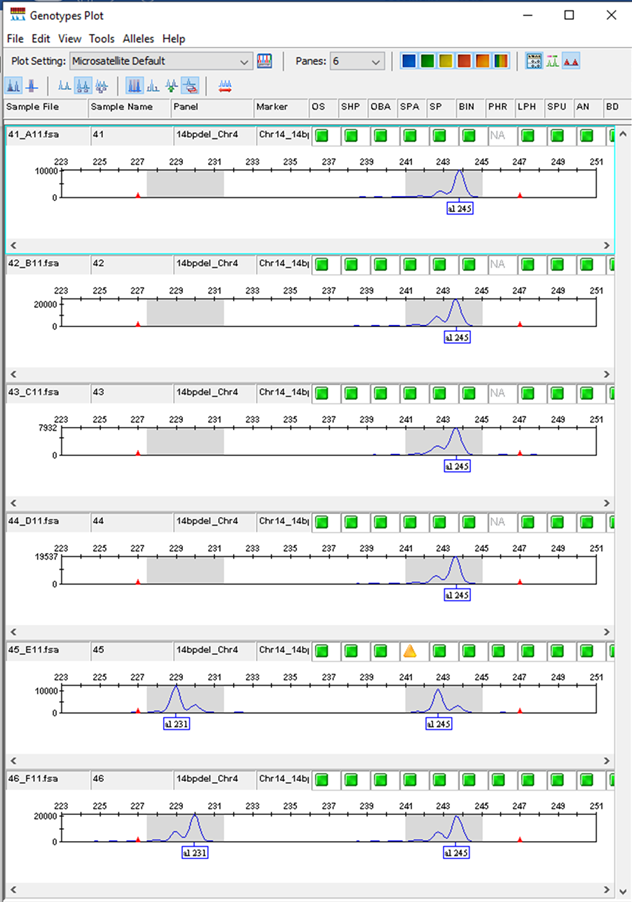


**Figure S11. Results of the fragment analysis on ABI3730 for the 14-bp deletion on chromosome 4.** The fragment analysis shows that F1-4 are homozygous wildtype and F5-6 are heterozygous for the 14-bp deletion.


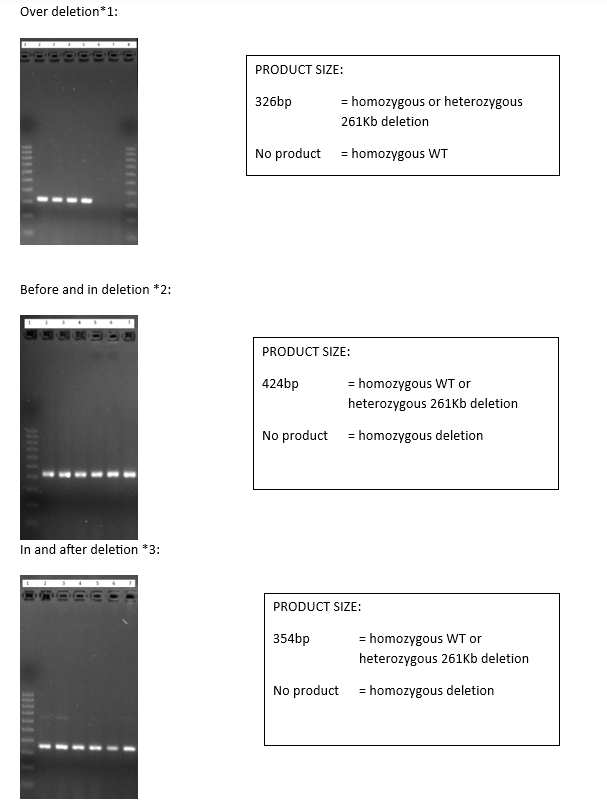


**Figure S12. Results of the agarose gel for testing on the 262-kb deletion.** The results show that F1-4 are heterozygous for the 261—kb deletion and F5-6 are homozygous wildtype.


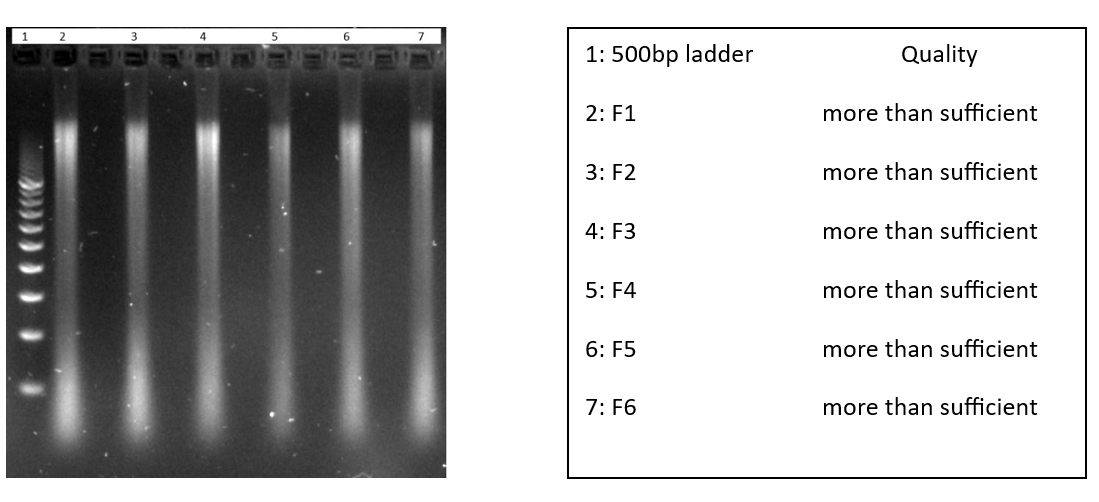


**Figure S13. Quality of gDNA for the 6 samples for PCR validation was checked on 0.8% agarose gel, 40 volt and for three hours.** All six samples show more than sufficient gDNA quality.
